# Stigma in Mental Health: The Status and Future Direction

**DOI:** 10.7759/cureus.85398

**Published:** 2025-06-05

**Authors:** Mustafa Habeb, Adela Magdalena Ciobanu, Mena Al-Ani, Richard Mottershead

**Affiliations:** 1 Department of Psychiatry, "Prof. Dr. Alexandru Obregia" Clinical Hospital of Psychiatry, Bucharest, ROU; 2 Department of Nursing, College of Health Sciences, University of Sharjah, Sharjah, ARE; 3 Department of Neuroscience, Discipline of Psychiatry, Faculty of Medicine, "Carol Davila" University of Medicine and Pharmacy, Bucharest, ROU; 4 Department of Developmental Biology and Cancer, University College London, Great Ormond Street Institute of Child Health, London, GBR

**Keywords:** psychiatry and mental health, public mental health, stigma against mental health issues, stigma in healthcare, stigma

## Abstract

It is well known that stigma significantly delays access to timely and appropriate mental healthcare, which then eventually causes a delay in achieving ideal health outcomes. It is distinguished by negative stereotypes, prejudice, and discrimination. It is relatively common in psychiatric care and noticeably linked to poor mental health, delayed availability of medical care and therapy, high morbidity, low quality of life, and suicide. The purpose of this research is to address the current situation of stigma in mental health. Based on Preferred Reporting Items for Systematic Reviews and Meta-Analyses (PRISMA) guidelines, PubMed, Medline, PsycInfo, and Social Science were searched from 1995 to April 2024. Abstracts of articles were then reviewed for relevance. Most studies used "mental illness" as a related term. This review shows that stigma in mental health is seen almost everywhere and could be a significant barrier for patients to seek medical help. In conclusion, stigma around mental health remains a significant barrier to effective care. A multidisciplinary approach is essential to effectively address stigma. Current efforts from governmental and non-governmental organizations are insufficient on their own. This can be done by implementing targeted awareness initiatives to change public perceptions, developing designated courses and workshops for patients, families, and community members, and launching coordinated campaigns involving various sectors (health, education, and media) to promote mental health literacy.

## Introduction and background

The term "stigma" is derived from the ancient Greek language, which means a symbol of shame, humiliation, and devaluation. It used to be a label for slaves and criminals in old times. Additionally, stigma is defined as the attitude by which the reaction of some people toward others could destroy their confidence and identity [1].

The presence of dysfunctional, irrational, and unfair opinions, emotions, and feelings of an individual or a group of people toward mental health patients generally represents stigmatization. Such patients may be perceived as displaying unacceptable social or mental characteristics, which are experienced by those who then stigmatize during social interaction with these individuals [2].

Nowhere in a community or culture do mentally ill people have the same societal value, acceptance, indulgence, and inclusiveness as people without a mental illness [3].

It is worth discovering more about the emotions surrounding stigma and how they are linked to joint attention to thoughts, feelings, intentions, and motives. Scheff argued that emotions are the fundamental foundation of social life; they play an important role in communication and connection skills. They are the viability of social structure and the cultural symbol system. Stigma originates from an imbalance in the status of emotions and feelings in normal social connections that require a balance between proximity and distance [4].

Historically, patients with mental health conditions such as depression, autism, and schizophrenia used to be enslaved or incriminated. They suffered torture, imprisonment, or even death. Mental illness was believed to be God's punishment during the medieval era; those patients used to be labeled as controlled by Satan, and fire was the main treatment for purification. Patients with mental illnesses were eventually released from cuffs and chains and admitted to specialized mental health facilities in the 17th century. Unfortunately, during the Nazi era in Germany, stigmatization and discrimination peaked at an unprecedented level, leading to the murder or castration of hundreds of thousands of mentally ill people [5]. Despite advances in modern societies, mentally ill people still experience difficulties in employment, education, housing, social inclusion, access to basic healthcare services, and marriages, and have complicated dynamics in relationships. For those with mental health issues, these are the real origins of social determinants of health [6].

Living with a mental illness carries a stigma that patients have described to be worse than the illness itself. Stronger forms of stigma usually create social anxiety or social phobia, which increases the burden on mental health patients. The primary defining characteristic of stigma is a largely ego-driven fear of what others would think of a person's behavior, thought, appearance, or speech. This results in the person being afraid to do or say something and wanting to avoid a negative reaction and being laughed at, insulted, criticized, or even rejected. A stigmatized person may choose to avoid social situations instead, leading to social withdrawal and deepening feelings of exclusiveness and isolation. These then worsen the symptoms, lower the likelihood of receiving treatment, and alter the recovery of people with mental illnesses. People with mental illness would eventually accept the suffering of mental distress without seeking help, rather than facing the risk of stigma, discrimination, labeling, classifying, and ostracization [3].

It is worth linking stigma with psychosocial disability, which usually arises in people who experience long-term mental distress and encounter stigma and discrimination, which are the main barriers that hold them back from fully participating in their society and eventually strengthen and deepen the feeling of being a burden on themselves and others [4].

Principally, stigma is divided into three levels: cognitive, emotional, and behavioral. These levels help in differentiating between common stereotypes, bias, and discrimination. Stereotypes are ideas and attitudes held toward members of specific groups, such as ethnicity, race, color, or religion. In terms of stigma in mental health, the most significant stereotypes assume laziness, difficulty, unintelligence, irresponsibility, dangerousness, violence (subtle stigma), unpredictability, and unreliability. Stigmatized mental health persons are often subjected to different types of abuse, such as emotional, physical, or sexual. Stigma differs from one mental illness to another. For instance, patients with schizophrenia and psychosis are rated the most dangerous compared to those with major depression or drug and alcohol dependence [5]. These stereotypes create generalizations rather than specific response patterns. Stigma might stem from a lack of understanding about mental health, as well as a lack of acceptance that mental health disorders exist. These disorders are just like any other physical health conditions, and both are classified, studied, and treated by evidence-based medicine. Furthermore, mental health conditions are also not the choice of patients; they are a result of multifactorial psychopathological interconnectedness. Unfortunately, some people may even avoid making friends or living with individuals with mental health issues, making mentally ill people more isolated and left alone with limited accommodation options [6,7].

Stigma could take the form of thoughts, beliefs, impressions, and judgments with no certain actions. Discrimination could take the form of negative verbal comments or, to some extent, could be developed into physical abuse or assault [8]. The severity of stigma differs from one region to another, and what seems to be the explanation of causation and the psychopathological understanding of mental disease in that area. For instance, in some Middle Eastern and African countries, they tend to link the etiology of mental illness with religious and magical causes. This even affects academia. It has been stated that 25% of low- and middle-income nations seem to lack mental health researchers, while an additional quarter have fewer than five researchers in this area [9]. Mental health researchers in low- and middle-income nations face inadequate funding and limited access to resources such as research networks, fellowships, technical assistance, or well-stocked libraries. Some initiatives are crucial in enhancing the community's awareness and acceptance of individuals with mental illness, including disseminating information, conducting campaigns, supporting clinical research, and encouraging extensive involvement of experts in mass media awareness programs [10].

Despite growing research on mental health stigma, key gaps persist in the literature. Notably, few studies explore stigma among mental health professionals themselves and its impact on patient care [9]. There is also a lack of culturally tailored interventions, limiting effectiveness in non-Western settings. Furthermore, the long-term effectiveness of anti-stigma programs remains under-evaluated. Finally, limited research links stigma directly to treatment outcomes, highlighting the need for longitudinal studies [10]. Addressing these gaps is essential for advancing effective stigma reduction strategies.

To our knowledge, there is currently a sparse literature review that addresses stigma in mental health illness and the lack of practical intervention to reduce it, which makes our review crucial. Therefore, we conducted this review to address and understand how common stigma is in mental health, determine the associated stigma with different mental health conditions, and present recommendations for decreasing the stigma toward individuals with mental issues and promoting research in this field.

## Review

Method

Based on Preferred Reporting Items for Systematic Reviews and Meta-Analyses (PRISMA) guidelines, PubMed, Medline, PsycInfo, and Social Science were searched from 1995 to April 2024. We conducted our search using the following keywords: stigma, mental illness, mental health, self-stigma, public stigma, discrimination, mental disorders, healthcare attitudes, and beliefs. A total of 1,532 articles were initially found using these search terms. One hundred article abstracts were then examined for applicability and chosen to extract data. Peer-reviewed journal publications were selected based on inclusion and exclusion criteria.

Inclusion Criteria

We included peer-reviewed articles, original research, recently published articles (last five years), and articles published in the English language.

Exclusion Criteria

Non-peer-reviewed literature, studies not focused on stigma, non-human studies, non-English publications, and duplicate data were excluded.

After removing duplicates, relevant articles were selected through a three-step process: screening of the titles and abstracts, exclusion of irrelevant articles, and thorough full-text examination of the selected articles to confirm relevance. Three independent reviewers conducted the article screening process, and any discrepancies were resolved through consultation and consensus.

Size of the problem

Surprisingly, a 2001 World Health Organization (WHO) report revealed that over 25% of people globally experience mental or behavioral disorders at some point in their lives. By 2020, mental and behavioral health problems accounted for 15% of the global disease burden, surpassing the estimate of 12% [11].

Stigma remains a major obstacle to receiving psychiatric care in various cultures. It causes delayed diagnosis and treatment-seeking behaviors, lower life quality, and a higher chance of discrimination and social exclusion [12]. Moreover, the stigma associated with mental illness frequently overlaps with stigma related to gender, race, and socioeconomic status. Furthermore, it creates further marginalization for a vulnerable population and makes it difficult to give equitable, culturally sensitive, and effectively delivered psychiatric care to those who suffer from mental illness and are in real need of intervention. Research has indicated that stigma associated with mental illness is widespread across cultural contexts and different countries, harming mental health diagnosis, treatment, and management that is deeply ingrained and reflected in traditions, attitudes, and beliefs [13-16].

Types of mental health stigma

Stigma is defined as any quality or trait that causes someone to be devalued, tarnished, shamed, or discredited. Nonetheless, the majority of authors fully agree with Goffman's definition, which accepts that the majority of stigma's constituents include discrimination, marginalization, labeling, stereotyping, social isolation, prejudice, rejection, ignorance, status loss, low self-efficacy, low self-esteem, and social isolation [16].

For instance, self-stigma refers to a person's negative attitude toward their mental illness and is also known as internalized stigma [15-17]. Relevant low outcomes, including not getting treatment, increased risk of substance abuse, feeling powerless, poor parenting, truancy, delinquency, low self-efficacy, and having a lower quality of life, have all been linked to self-stigma [10-19]. Public stigma describes the adverse impressions that the general public has toward people with mental illnesses, which are frequently rooted in prejudice, fear, and misconceptions. In addition, perceived stigma is defined as personal beliefs about how others view mental illness. Studies have largely indicated the significant influence of societal stigmas, such as discrimination, in public agencies and workplaces [7,15,16]. Professional stigma arises when medical personnel have stigmatizing beliefs about their patients, which are usually motivated by misinformation or fear about the mental illness. It can also happen when medical personnel face stigma from the general public or other medical professionals due to the nature of their work with stigmatized mentally ill people [15]. Professional stigma is especially concerning because it has the potential to influence the care and medical attention that a person with mental illness receives, including that for physical illness, hence affecting their health and general well-being. The policies or culture of an organization toward stigmatized people, such as those with mental health issues, is referred to as institutional stigma. In addition to being firmly ingrained in society, such stigma may also be addressed and mitigated by governmental regulations, professional standards, and legal frameworks [20].

Regarding mental health services, stigma has been noted as a significant problem for both clients and relatives. It can lead to a person's condition worsening or lead to repeat re-admissions because it makes receiving appropriate, timely, professional medical and psychological care difficult [21]. Additionally, the negative effects of stigma are considerably severe, as some patients compare the prejudice and stigma they experience to their disorder's symptoms, and they consider it a burden on their personal and professional lives. Stigma affects professionals who work in mental healthcare settings and the families and relatives of patients with mental health conditions [22,23].

As for interpersonal stigmas related to mental health issues, it has been discovered that institutional policies and other societal factors distinguish individuals with mental health issues, causing them to be denied access to resources and opportunities [24]. In comparison to the general population, individuals with mental health disorders experience a lower quality of life in addition to a higher incidence of illness, different types of abuse, neglect, suicide, and death. The social stigma that shadows mental illness persists in patients despite the recent advances in acceptance and understanding of mental illness in the field. This is ultimately because society still lacks awareness of mental illness [25].

Mental health stigma in Romania (the status)

Winkler et al. found that stigma in Eastern European countries appears to be greater than in other European nations. It was reported that various negative effects of stigma, such as delay in seeking help and acceptance of treatment, are widely prevalent in that region. Moreover, fewer opportunities for education, employment, and social contacts, or difficulties in finding accommodation for mentally ill patients are also evident in those countries. Although hampered in adjusting to the European Union standards since 2007, the legislations regarding mental health in Romania were altered and updated more on a theoretical but not practical basis [26]. Study results demonstrated that self-stigma is less frequent in the Romanian community compared to Malta, Croatia, and Lithuania, but higher than in the Swedish community [27].

Intriguingly, four major projects were carried out by local mental health centers in various parts of Romania between 2007 and 2008. They sought to raise community awareness of mental health issues and address the pressing need to change the way the public views those who suffer from mental illness [28].

According to a different study, Romania appears to have a lower correlation between public stigma and self-stigma than other nations like the USA, Australia, Canada, and the UAE, which may be a good place to accept patients with mental health issues [29].

What are the main sources of stigma in healthcare?

Stigma occurs on multiple levels simultaneously: intrapersonal (e.g., self-stigmatization), interpersonal (e.g., relationships with others), and structural (e.g., discriminatory and/or exclusionary policies, laws, and systems). It has also been reported that only significant social groups could be stigmatized [20]. In several countries, stigma affects staff working in mental healthcare settings, which could cause staff shortages in mental health systems [6].

An interesting study has identified certain issues that have several impacts on the quality of care for patients with mental illness, as well as the ability to connect to stigma in healthcare. These were referred to as "key learning needs," recognizing specific concerns that can be resolved with focused efforts. Studies involving Canadian healthcare professionals confirm that the stigma associated with the willingness to disclose or seek treatment for mental illness is higher than that associated with other aspects, like negative attitudes and the requirement for social distancing [30].

Consequences of stigma

The consequences of stigma create multidimensional barriers, such as delays in seeking help, discontinuation of treatment, suboptimal therapeutic relationships, patient safety concerns, and poorer quality of mental and physical care [20]. For example, expected stigma from healthcare providers has been identified as a factor in people's reluctance to seek help for mental illness [31]. Also, impairment of the relationship between the patient and the service provider and premature termination of treatment are among the consequences. A survey found that 79% reported first-hand experiences of discrimination against patients in general, and 53% observed other healthcare providers discriminate against a psychiatric patient [32]. Another Canadian study reported that procedural culture, team attitudes, and the recognized marginalization of mental health patients as a result of stigmatization all contribute to stigma as an obstacle to patient care [33].

People with mental health conditions commonly report difficulties getting their physical care needs met, including not having their symptoms taken truthfully and seriously when looking for care for non-mental well-being concerns [34]. This usually occurs largely through diagnostic and treatment overshadowing, whereby physical symptoms are misattributed to a patient's mental illness, causing delays in diagnoses and treatment options [35].

We believe that it is imperative to thoroughly assess the implications of stigma within this specific framework. Indicators such as an initial hesitance to seek assistance could potentially cause a decline in productivity, subsequently reinforcing prevailing stereotypes and eliciting further stigmatization from colleagues. This, in turn, amplifies the difficulty in seeking help.

Ways to reduce stigma surrounding mental illness

There is no single tailored intervention to tackle stigma in mental health. However, there are several alternate ways that could be gathered and implemented to reduce stigma in mental health. Also, there is a real need for multidimensional interventions from governmental departments such as justice, health, and social services, and active participation of stakeholders.

Crucial components that could reduce stigma in healthcare contexts have also been recognized. These include training courses that support healthcare [30]. Furthermore, social contact focuses on listening to people who witness stigma, as well as their involvement within the healthcare system, which could be a good approach for interprofessional educational methodologies to reduce stigma in healthcare [36]. Social contact remains a strong approach to disconfirming stereotypes, diminishing anxiety, heightening empathy, and improving understanding of recovery. Additionally, skills-based training also holds potential value as a model for decreasing stigma. It focuses on improving confidence, comfort, and understanding of mental illnesses [30].

Furthermore, there is a need for establishing community-based mental health services that are recovery-oriented and public-centered to involve patients and their families in discussions. This is in addition to the need for regular monitoring and evaluation of mental health services to ensure that up-to-date practices and protocols are in place. Moreover, high-standard training for mental healthcare professionals to address stigma empowers them to build capacity and protect their rights by stipulating regulations and laws. It is important to shed light on several countries that run campaigns such as "Time to Change Anti-Stigma Campaign" in England, "Beyond Blue," a mental health literacy program in Australia, and "Opening Minds," a contact-based educational provision in Canada. These campaigns aim to increase public awareness, fund research and evaluation, and empower mental health training programs through social media [37].

## Conclusions

One of the most significant findings in this review is that stigma is not a local issue: it is an international phenomenon that affects almost all communities worldwide. Although much research has been performed in this field, there is a lack of practical intervention. As we previously mentioned, there is no single tailored intervention to tackle mental health stigma. There is a real need for multidimensional collaboration between governmental and non-governmental organizations to effectively address the stigma associated with mental health patients. As the next step, an appropriate effort must be made to increase the awareness of society regarding the rights of mental health patients. This can be done through campaigns and social media to establish the rights of individuals affected by mental health stigmatization and empower them. Moreover, establishing available, accessible, and cost-effective mental health community services for stigmatized patients is crucial to mitigate the barriers they encounter in seeking such services. All these aspects should be considered as keys to understanding, assisting, retaining, and treating such patients within the mental health system.
